# Effects of physicochemical parameters on volatile sulphur compound formation from l-methionine catabolism by non-growing cells of *Kluyveromyces lactis*

**DOI:** 10.1186/s13568-018-0639-7

**Published:** 2018-07-03

**Authors:** Yuyun Lu, Margarete Nawrath, Jingcan Sun, Shao-Quan Liu

**Affiliations:** 1 Food Science and Technology Program, Department of Chemistry, National University of Singapore, Science Drive 3, Singapore, 117543 Singapore; 2 National University of Singapore (Suzhou) Research Institute, 377 Lin Quan Street, Suzhou Industrial Park, Suzhou, Jiangsu 215123 China

**Keywords:** *Kluyveromyces lactis*, l-Methionine, Volatile sulphur compounds, Bio-flavors

## Abstract

The present study investigated for the first time the effects of various physicochemical parameters on the production of volatile sulphur compounds (VSCs) by non-growing cells of *Kluyveromyces lactis* supplemented with l-methionine. The results showed that the production of VSCs positively correlated with the cell biomass, but it seemed that no clear relationship with l-methionine concentration existed. Temperature and pH significantly affected the formation of VSCs with more production at 30 °C and pH 5, respectively. Nitrogen supplementation (in the form of diammonium phosphate, DAP) repressed the production of VSCs. It is interesting to note that DAP and yeast extract supplementation induced the production of methional, but not Mn^2+^ supplementation. The presence of Mn^2+^ improved the production of methionol and dimethyl disulphide, but inhibited the formation of *S*-methyl thioacetate. The study indicated that optimization of physicochemical conditions and media composition would be crucial for producing l-methionine-derived VSC bioflavor.

**Electronic supplementary material:**

The online version of this article (10.1186/s13568-018-0639-7) contains supplementary material, which is available to authorized users.

## Introduction

Volatile sulphur compounds (VSCs) are responsible for the intensive odor of vegetables such as cabbage, onions and garlic. They normally have distinctive desirable or undesirable odor qualities and are frequently found in fermented products like wine, cheese or beer where they significantly impact sensorial attributes due to their extremely low odor detection thresholds ranging from ng/L to µg/L (Mestres et al. 2000; Ugliano and Henschke 2009; McGorrin 2011).

The sulfur-containing amino acids such as l-cysteine and l-methionine are the main precursors of VSCs in fermented products. Methanethiol is the first intermediate from l-methionine metabolism by yeasts and is also regarded as a precursor of many other VSCs (Hanniffy et al. 2009). Methanethiol was found in various foods including blue cheese and alcoholic beverages with contribution of a putrid or cooked cabbage-like flavor (Gonda et al. 2013; Ochiai et al. 2012; Hazelwood et al. 2008; Landaud et al. 2008). Due to its high oxidation sensitivity, methanethiol is usually rapidly converted into dimethyl disulfide (DMDS) and dimethyl trisulfide (DMTS) (Chin and Lindsay 1994). Further, DMDS and DMTS could be transformed back into methanethiol during fermentation. DMDS could contribute to a garlicky and onion-like odor, which is of benefit to the flavor of some fermented foods such as *Tuber melanosporum* (Xiao et al. 2015) and cheese (Fuchsmann et al. 2015).

Methional is also considered as a catabolite from l-methionine metabolism by cheese-ripening bacteria or yeasts with a characteristic odor of boiled potatoes. In addition, methional is known for its light-induced off-flavor in milk and is first formed via Strecker degradation, due to it being unstable, it could decompose to mercaptomethane, and finally to DMDS (Ahn et al. 2016). Seow et al. (2010) reported that methional could also be produced from l-methionine metabolism by yeasts in coconut cream.

*S*-Methyl thioesters can be formed via enzymatically from l-methionine metabolism by cheese-ripening yeasts (Martínez-Cuesta et al. 2013). The formation of *S*-methyl thioesters is relatively dependent on pH and temperature in the presence of methanethiol and short-chain fatty acids which need to be activated by CoA-SH (Helinck et al. 2000; Martínez-Cuesta et al. 2013). *S*-Methyl thioacetate (MTA), which has a boiled cauliflower-like aroma, is the most prevalent thioester found in cheeses. MTA was most frequently associated with cabbage and cheesy odors, but it is an unsuspected odor compound in fresh strawberries (Schulbach et al. 2004).

Various microorganisms have been reported to possess the VSC production capacity including bacteria (e.g. *Brevibacterium linens* or *Lactococcus lactis*) and yeasts (e.g. *Geotrichum candidum*, *Yarrowia lipolytica* and *Kluyveromyces lactis*) in cheese and wine production (Arfi et al. 2002, 2003; Spinnler et al. 2001; Cernat-Bondar et al. 2005; Kagkli et al. 2006a, b). Most of VSCs are detected as key aroma compounds and contribute to the overall flavor of cheeses. However, they could also contribute to off-flavors when the concentration is in excess, especially in wine or beer (Landaud et al. 2008).

Previous studies showed that yeasts are able to metabolize l-methionine into VSCs via the Ehrlich pathway (McGorrin 2011; Seow et al. 2010; Tan et al. 2012). *K. lactis* has received increasing attention in industrial biotechnology over the last decade due to its distinctive fermentative capabilities. This yeast plays an important role in formation of VSCs and is mainly involved in cheese aromatization during the ripening process. Furthermore, *K. lactis* is unable to grow under anaerobic conditions and an oxygen limitation will cause a significant decrease of its growth rate (Merico et al. 2009). In contrast to other yeasts, *K. lactis* has the ability to utilize lactose due to the capacity of producing *β*-galactosidase (Rodicio and Heinisch 2013). In addition, *K. lactis* has been identified as a cheese ripening yeast that has a great ability in VSC biogenesis owing to its genetic makeup (Cholet et al. 2007).

Recently, non-growing cells as a whole-cell biocatalysis system have been used to improve the biotechnological process (Julsing et al. 2012). Non-growing cells are metabolically active microorganisms which are applied to a synthetic medium supplemented with a product precursor. This procedure is based on energy limitation to inhibit biomass production and increase product yield by enhancing the efficiency of metabolization (Julsing et al. 2012). Using non-growing cells of *K. lactis*, VSC biosynthesis can be more efficient and may be more economically viable. The control of metabolic processes of l-methionine provides biotechnological ways to produce bioflavor product and at the same time, addressing consumer preferences for natural ingredients (Rodicio and Heinisch 2013). In this study, the whole-cell-based catabolism of l-methionine was conducted with the objectives of understanding l-methionine metabolism and VSC formation by *K. lactis* under various physicochemical conditions. The formation of VSCs was examined in a model system consisting of non-growing cells of *K. lactis* and l-methionine.

## Materials and methods

### Reagents and standards

Malt extract, yeast extract, bacteriological peptone and potato dextrose agar (PDA) were purchased from Oxoid (Hamphire, England). Manganese (II) chloride tetrahydrate (MnCl_2_·4H_2_O) and HCl were purchased from Merck KGaA (Darmstadt, Germany). Sucrose was purchased from Reckitt Benckiser (Glucolin^®^, Petaling Jaya, Malaysia). Sodium phosphate monobasic dihydrate (NaH_2_PO_4_·2H_2_O), sodium phosphate dibasic anhydrous (Na_2_HPO_4_), l-methionine (≥ 99%, non-animal source) and diammonium phosphate (DAP, purity ≥ 98%) were purchased from Sigma-Aldrich (Unterhaching, Germany).

### Culture preparation and cell harvesting

*Kluyveromyces lactis* KL71 was obtained from Danisco Singapore Pte Ltd (Singapore). The freeze-dried yeast was (with shaking, 80 rpm) cultured at 25 °C for 24 h in sterile broth (pH 5.0, 1 M HCl) containing 2% (w/v) glucose, 0.25% (w/v) yeast extract, 0.25% (w/v) malt extract and 0.25% (w/v) bacteriological peptone. After that, the pure culture was aliquoted into 1-mL sterile tubes and stored at − 80 °C. The pre-culture of *K. lactis* KL71 was prepared by inoculating 5% (v/v) of a pure culture in the sterilized broth (100 mL) and incubated at 30 °C for 24 h under sterile conditions with shaking continuously at 80 rpm in water bath. The obtained pre-culture was further inoculated into the sterile broth (3%, 3 L). The sterile broth was the same as above except the amount of glucose was increased to 3% (w/v).

The propagated yeast cells were centrifuged in sterile 50-mL PP tubes (Greiner Bio-one, Germany) at 4700 rpm for 15 min (Centrifuge 5810R, Eppendorf AG, Hamburg, Germany). The supernatant was discarded and the obtained yeast cells were washed twice with a sterile sodium phosphate buffer (pH 5, 100 mM), and then centrifuged at 8000 rpm for 10 min at 4 °C. The supernatant was decanted and the washed cells were re-suspended in 100 mL of the phosphate buffer (pH 5, 100 mM) and stored overnight (< 24 h) at 4 °C.

### Whole-cell biocatalysis conditions and procedures

A total of eight parameters were investigated at three levels. For investigations on physicochemical parameters (biomass, l-methionine concentration, agitation rate/aeration, temperature and pH), the factor level which exhibited the highest methionol production was selected for subsequent treatments. For parameters of the supplementation with nitrogen (DAP), yeast extract and Mn^2+^, they were investigated individually by using the selected physicochemical parameters in triplicate.

Non-growth media consisted of yeast cells and l-methionine as shown in Table 1 and were filled up with sterile 100 mM phosphate buffer to a total volume of 100 mL. Whole-cell biocatalysis was conducted in sterilized 250-mL Schott glass bottles with screw caps (Schott AG, Delligsen, Germany).

**Table 1:** Factor levels investigated for each parameter

Run	Parameter	Factor levels		
		Low	Medium	High
1	Biomass (OD_600_ value)	2	4	6
2	l-Methionine concentration (w/v)	0.1%	0.5%	1.0%
3	Agitation rate (rpm)	0	80	120
4	Temperature (°C)	25	30	38
5	pH (100 mM phosphate buffer)	4	5	6
6	Nitrogen supplementation (w/v)	0%	0.1%	0.3%
7	Yeast extract (w/v)	0%	0.1%	0.3%
8	Mn^2+^ supplementation (mM)	0	1	10

#6: nitrogen = diammonium phosphate

To investigate the biomass, the optical density (OD) was measured with a UV–Vis spectrophotometer (UVmini-1240, Shimadzu, Kyoto, Japan) at 600 nm. The reaction mixtures comprised of 0.5% l-methionine in 100 mM phosphate buffer at pH 5 and different amounts of *K. lactis* 71 with OD_600_ of 2, 4 and 6 were incubated aerobic for 48 h in water bath at 30 °C with 80 rpm.

An l-methionine stock solution (4%, w/v) was prepared in 100 mM sodium phosphate buffer and was filtered by using sterile Acrodisc^®^ syringe filters with a Supor^®^ membrane (0.20-µm, Pall Corp., Port Washington, USA) before adding aseptically to the non-growth media to achieve the final cell biomass (Table 1).

Agitation and temperature were controlled by using shaking water bath (SW22, Julabo GmbH, Seelbach, Germany). Without agitation, Schott bottles with media were screwed tightly to create a near anaerobic condition. At 80 and 120 rpm, the caps were screwed loosely to ensure aeration. The pH of media was adjusted by varying the amounts of NaH_2_PO_4_ and Na_2_HPO_4_. Additionally, a pH meter (Metrohm Ltd., Herisau, Switzerland) was used for verification.

For supplemented treatments, the preparation was carried out as described above. Diammonium phosphate was used as the nitrogen source. Yeast extract was identical to the one used in broth. Both stock solutions were filter sterilized. Manganese (II) chloride tetrahydrate as the Mn^2+^ source was diluted in 100 mM phosphate buffer and sterilized in an autoclave (Hirayama HVE-50 Hiclave, Tokyo, Japan) at 121 °C for 20 min.

Samples (6 mL) were taken at 0, 2, 8, 24 and 48 h. The whole-cell biocatalysis was stopped by removing yeast cells via centrifugation (8000 rpm) at 4 °C for 10 min, and the pH of the supernatant was adjusted to 2.5 by adding 1.0 M HCl before volatile analysis. All samples were stored at − 80 °C before analysis.

### Yeast cell quantification

The viable cells in initial non-growth media (0 h) and at the end of incubation (48 h) were obtained by spread plating on potato dextrose agar (PDA). Plates were incubated at 25 °C for 48 h (Sanyo Incubator, Illinois, USA). In addition, streaking was carried out to confirm the absence of contaminants of both the pre-culture and reaction media before and after incubation (0 and 48 h).

### Volatile sulfur compound analysis

All samples were analyzed to identify and semi-quantify volatile compounds using head space solid-phase micro extraction coupled with gas chromatography–mass spectrometry and flame ionization detection (HS-SPME–GC–MS–FID) as reported previously (Tan et al. 2012; Seow et al. 2010). The volatile sulfur compounds were identified by comparing the mass spectra with NIST 8.0 and Wiley 275 MS library.

### Statistical analysis

The mean values and standard deviations were calculated based on the data obtained from triplicate incubations. All data were subjected to one-way analysis of variance (ANOVA) to investigate the significant difference among factor levels and to determine optimal conditions for methionol and other VSC production. All tests of significance were conducted at a probability level of *P* value less than 0.05.

## Results

### Effect of physicochemical parameters on VSC production by non-growing cells of *K. lactis*

**Fig. 1:**
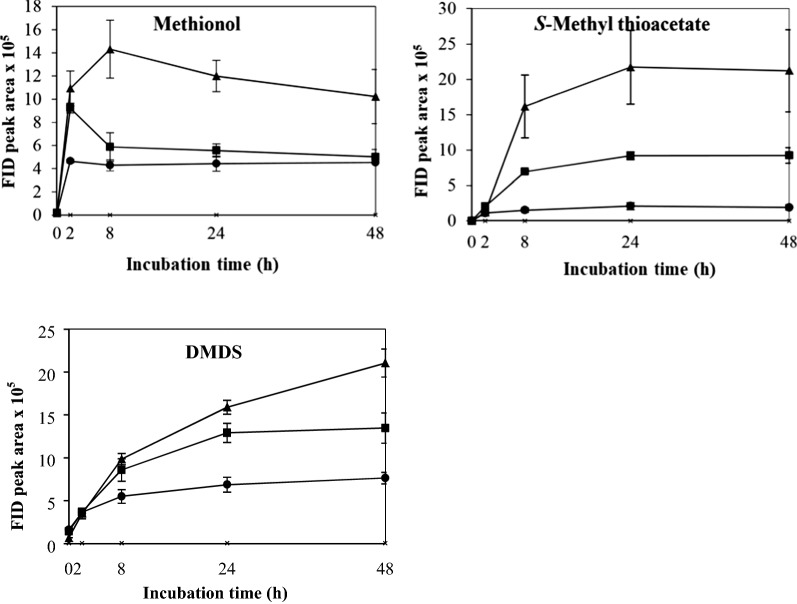
Kinetics of volatile sulphur compound production during 48 h’ incubation at OD_600_ levels of 2 (black circle), 4 (black square) and 6 (black up-pointing triangle)

**Fig. 2:**
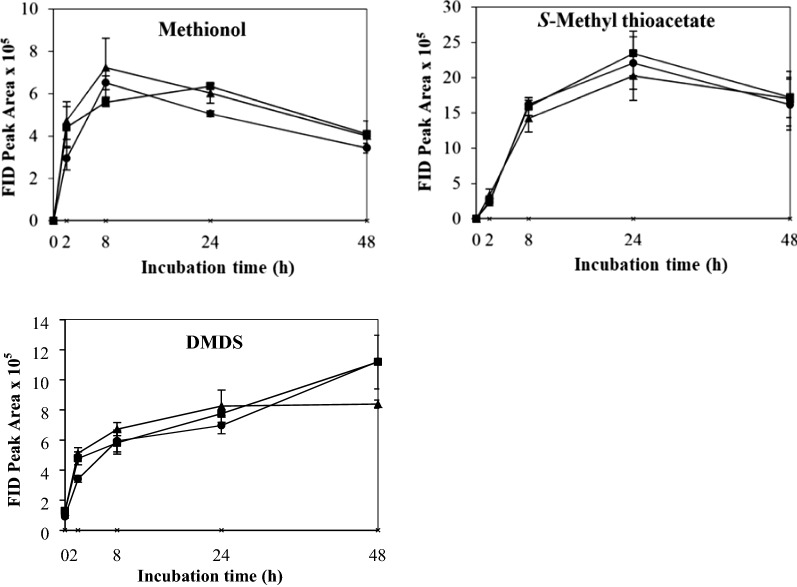
Kinetics of volatile sulphur compound production during 48 h’ incubation at l-methionine concentrations 0.1% (black circle), 0.5% (black square) and 1.0% (black up-pointing triangle)

**Fig. 3:**
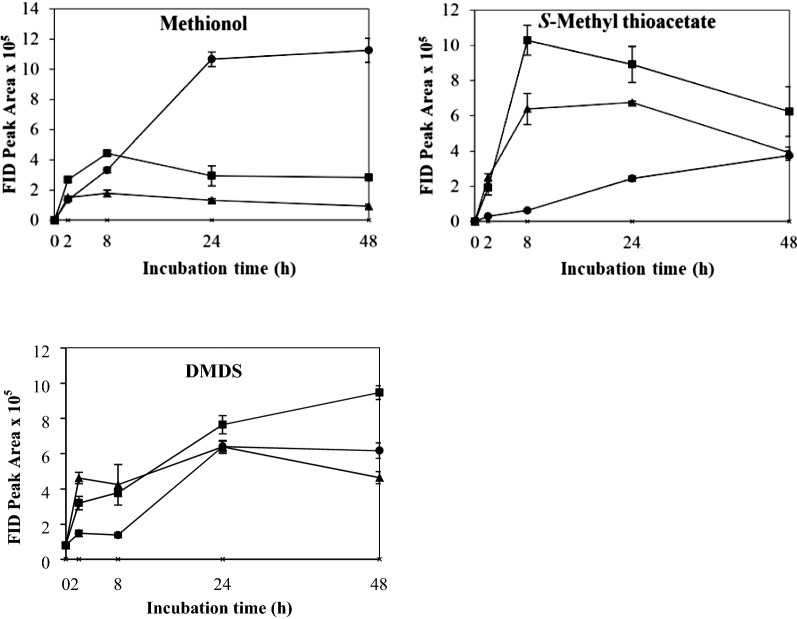
Kinetics of volatile sulphur compound production during 48 h’ incubation at agitation rates 0 rpm (black circle), 80 rpm (black square) and 120 rpm (black up-pointing triangle)

**Fig. 4:**
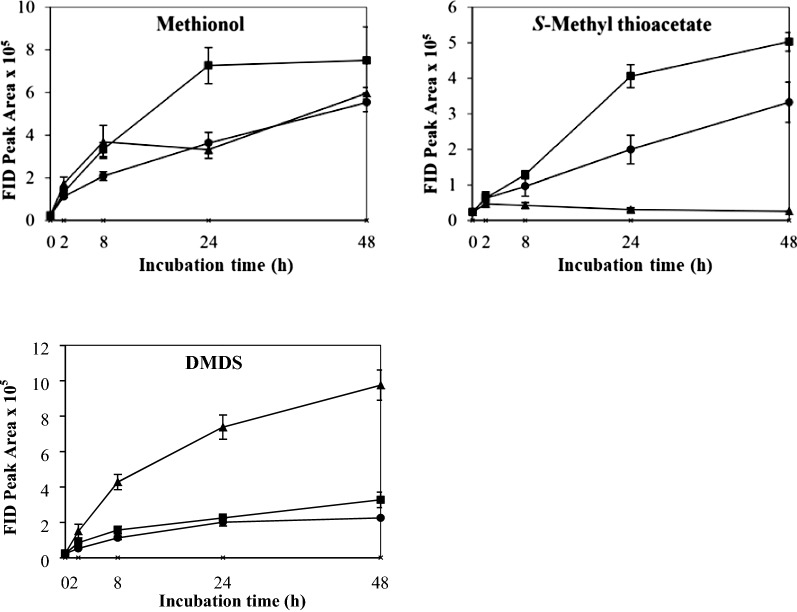
Kinetics of volatile sulphur compound production during 48 h’ incubation at temperatures 25 °C (black circle), 30 °C (black square) and 38 °C (black up-pointing triangle)

**Fig. 5:**
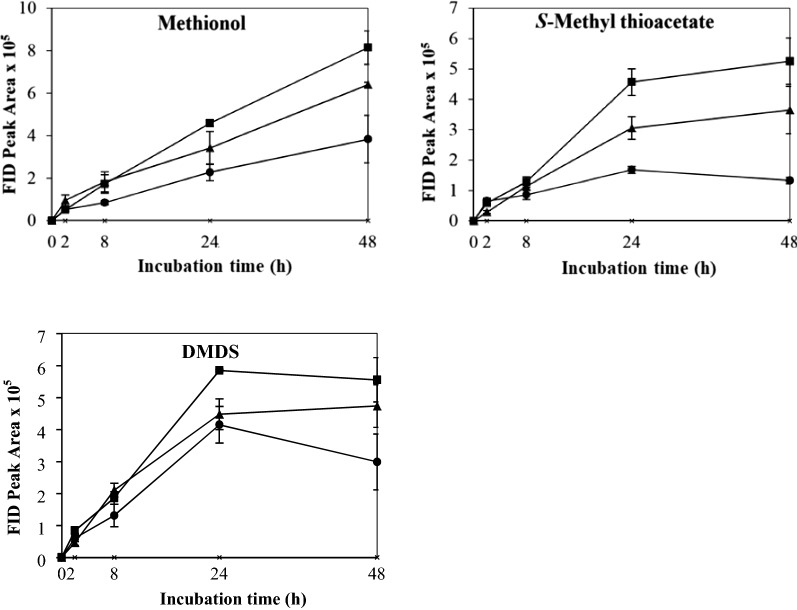
Kinetics of volatile sulphur compound production during 48 h’ incubation at pH 4 (black circle), 5 (black square) and 6 (black up-pointing triangle)

The control which contained yeast cells only in buffer showed no VSC production after 48 h’ incubation. The effects of different physicochemical parameters on kinetic changes of VSC production including methionol, MTA and DMDS are shown in Figs. 1, 2, 3, 4, 5. Other VSCs were not detected.

The effect of biomass on the production and kinetic changes of methionol, MTA and DMDS is shown in Fig. 1. The production of VSCs positively correlated with the biomass as measured as OD_600_. Methionol increased sharply and reached its maximum at 2 or 8 h and then decreased slightly or kept stable (Fig. 1). The production of methionol between 8 and 48 h was significantly higher at OD_600_ 6 than that at OD_600_ 4 and OD_600_ 2 (Fig. 1 and Additional file 1: Table S1). MTA increased gradually by 24 h and then declined slightly or remained stable (Fig. 1). Production of MTA at OD_600_ 4 and 6 was significantly higher than that at OD2, whereas no significant difference was found between OD_600_ 4 and OD_600_ 6 (Fig. 1 and Additional file 1: Table S1). DMDS increased gradually and kept stable except at OD_600_ 6, which increased continuously (Fig. 1). It is important to note that the higher the OD_600_ value, the significantly higher amount of DMDS was produced (Fig. 1 and Additional file 1: Table S1). Therefore, OD_600_ 6 was selected and used in subsequent treatments.

The effect of l-methionine concentration (0.1, 0.5 and 1.0%) on the production and kinetic changes of VSCs is shown in Fig. 2. All treatments followed similar kinetic changes with methionol increasing to its maximum at 8 h (0.1 and 1.0%) or 24 h (0.5%) and then declined onwards (Fig. 2). Similarly, the production of MTA increased gradually by 24 h and then declined in all treatments (Fig. 2). As for DMDS, it increased gradually but with relatively higher DMDS production at l-methionine concentration of 0.1 and 0.5% (Fig. 2). The production of DMDS may partially come from the oxidation of methanethiol released from the hydrolysis of MTA. It seemed that there was no significant effect of l-methionine concentration on VSC production among all treatments, except DMDS between 0.1 and 1.0% of l-methionine (Additional file 1: Table S1). Taking analytical variations into consideration, 1.0% l-methionine was selected and used in subsequent treatments.

The effect of aeration on the production and kinetic changes of VSCs is shown in Fig. 3 and the aeration seemed to decrease the formation of methionol. The production of methionol increased gradually by 24 h and then remained largely stable under static condition. The amount of methionol produced was significantly higher than that at 80 and 120 rpm, where methionol increased to the maximum by 8 h and then declined slightly (Fig. 3). The production of MTA increased first and then declined gradually with agitations (Fig. 3). The highest production of MTA was found at 80 rpm (Fig. 3) but there was no significant different in the final amount of MTA among the treatments (Additional file 1: Table S1). The production of DMDS followed similar trends except 120 rpm, which increased first by 24 h and then decreased slightly (Fig. 3). Significantly higher level of DMDS was found in samples treated at 80 rpm (Additional file 1: Table S1). Although the production of MTA and DMDS were improved with aeration, tight enclosure of bottles and static incubation were adopted in subsequent treatments due to the clearly positive effect on methionol formation.

The effect of temperature on the production and kinetic changes of VSCs is shown in Fig. 4. The production of methionol followed a similar trend with the highest production at 30 °C (Fig. 4 and Additional file 1: Table S1). Trace amounts of MTA were produced when incubated at 38 °C, while significantly higher levels were produced at 25 and 30 °C (Fig. 4 and Additional file 1: Table S1). In addition, relatively higher levels of MTA were produced at 30 °C than that at 25 °C but no significant difference. In contrast, the highest production of DMDS was found at 38 °C, which was significantly higher than that at 25 and 30 °C (Fig. 4 and Additional file 1: Table S1). Taking all VSCs into consideration, 30 °C was selected and used in subsequent treatments.

The effect of pH on the production and kinetic changes of VSCs is shown in Fig. 5. All VSCs followed similar trends where VSCs increased gradually (Fig. 5). The highest production of methionol, MTA and DMDS was found at pH 5.0, followed by pH 6.0 and pH 4.0 (Fig. 5 and Additional file 1: Table S1). These results indicated that the optimum pH for VSC formation was around 5.0, being consistent with the known optimum pH for yeast growth. Therefore, pH 5.0 was selected and used in subsequent treatments.

### Effects of nutrient supplementation on kinetic changes of VSCs by non-growing cells of *K. lactis*

In the following treatments, the selected parameters with OD_600_ 6, 1.0% l-methionine, static incubation, 30 °C and pH 5 were used to investigate the effects of specific nutrients on the production and kinetic changes of VSCs by non-growing cells of *K. lactis*.

**Fig. 6:**
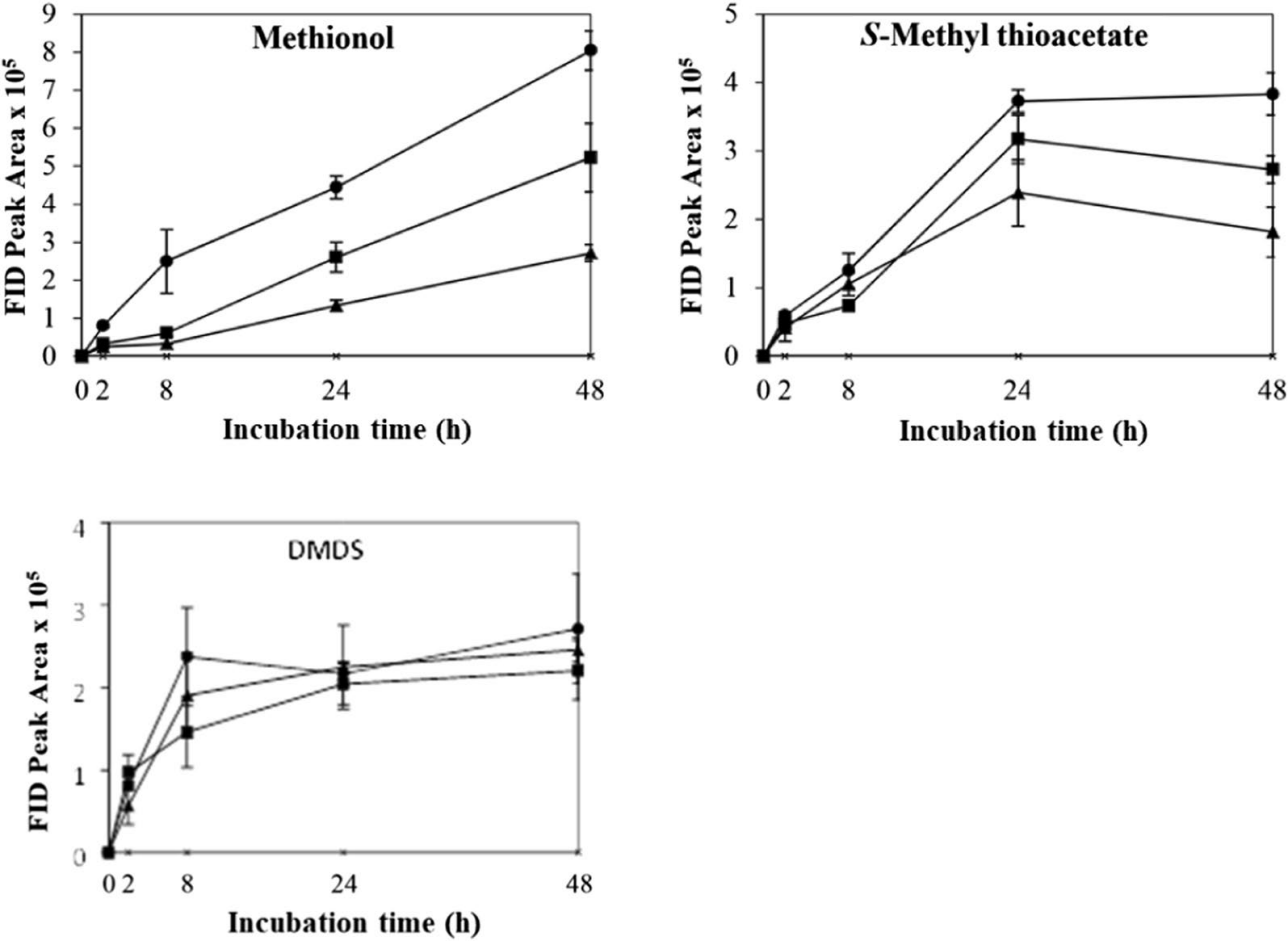
Kinetics of volatile sulphur compound production during 48 h’ incubation with nitrogen (diammonium phosphate) concentrations 0% (black circle), 0.1% (black square) and 0.3% (black up-pointing triangle)

The effect of nitrogen supplementation with DAP on the production and kinetic changes of VSCs is shown in Fig. 6. It seemed that nitrogen supplementation suppressed the production of VSCs with the control producing the highest level of methionol, MTA and DMDS, followed by 0.1 and 0.3% (Fig. 6). However, there is no significant difference in the final amount of DMDS among all treatments (Additional file 1: Table S1). It is interesting to note that trace levels of methional were detected in the treatments supplemented with DAP, though not statistically different (Additional file 1: Table S1).

**Fig. 7:**
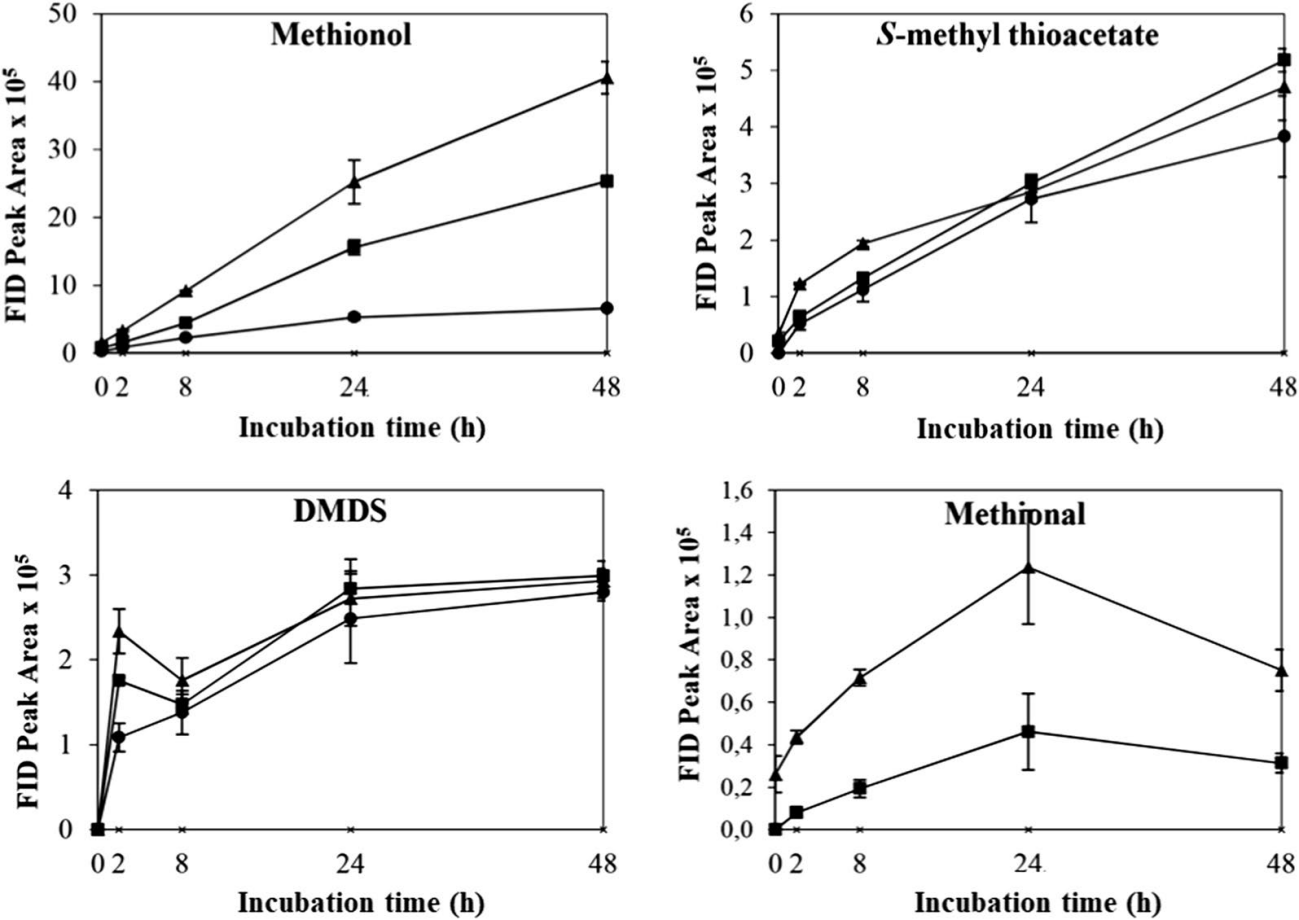
Kinetics of volatile sulphur compound production during 48 h’ incubation with yeast extract concentrations 0% (black circle), 0.1% (black square) and 0.3% (black up-pointing triangle)

The effect of yeast extract supplementation on the production and kinetic changes of VSCs is shown in Fig. 7. There is a significant impact of yeast extract on the production of methionol with 0.3% supplementation producing the highest level, followed by 0.1 and 0%, respectively (Fig. 7 and Additional file 1: Table S1). The formation of methionol in 0.3% supplementation is around 2 times (in terms of FID peak area) higher than 0.1% supplementation and around 6 times higher than that in the control (Fig. 7 and Additional file 1: Table S1). Although yeast extract supplementation did not significantly boost the production of MTA and DMDS (Additional file 1: Table S1), there was an evident tendency to increase. Yeast extract supplementation induced the formation of methional with 0.3% addition producing the highest level, followed by 0.1%; methional reached a maximum at 24 h, then declined. No methional was found in the control (0%).

**Fig. 8:**
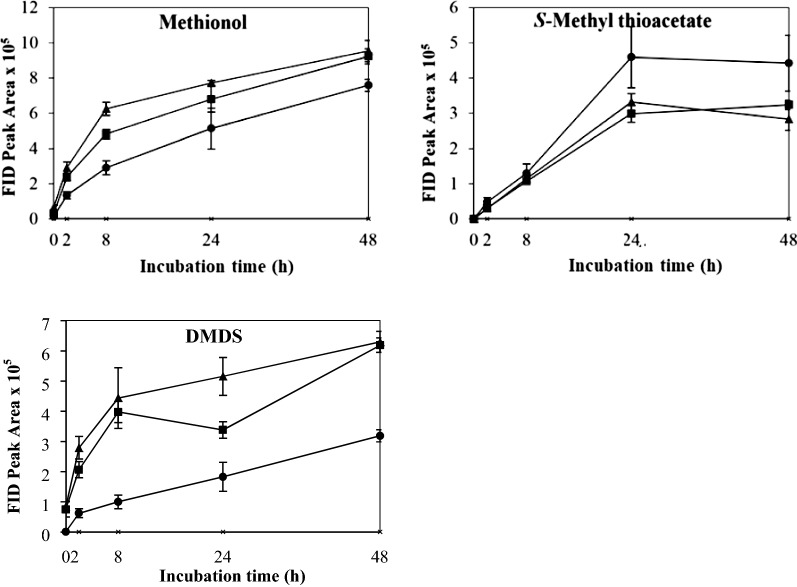
Kinetics of volatile sulphur compound production during 48 h’ incubation with Mn^2+^ concentrations 0 mM (black circle), 1 mM (black square) and 10 mM (black up-pointing triangle)

The effect of Mn^2+^ supplementation on the production and kinetic changes of VSCs is shown in Fig. 8. In the presence of Mn^2+^, more methionol was produced with more Mn^2+^ being added (Fig. 8 and Additional file 1: Table S1). However, Mn^2+^ reduced the formation of MTA with 10 mM producing the lowest amount (Fig. 8 and Additional file 1: Table S1). Mn^2+^ supplementation significantly increased the production of DMDS (Fig. 8 and Additional file 1: Table S1). In contrast to nitrogen and yeast extract supplementation, methional was not detected (Additional file 1: Table S1).

### Effects of investigated parameters on cell survivability

The yeast cell counts before and after incubation is summarized in Additional file 1: Table S2. The viable yeast cells were reduced after 48-h incubation in non-growth media except in treatments at 25 °C and yeast extract supplementation, where relatively higher cell counts were obtained but without significant difference relative to the respective initial cell counts (Additional file 1: Table S2). Significant decreases of viable yeast cells occurred in treatments at pH 4.0 and Mn^2+^ supplementation (Additional file 1: Table S2). In addition, at 38 °C, no viable cell counts were obtained.

## Discussion

### Effect of physicochemical parameters on VSC production by non-growing cells of *K. lactis*

The influence of several physicochemical parameters on the production and kinetic changes of VSCs via the metabolism of l-methionine was investigated using non-growing cells of *K. lactis* (Figs. 1, 2, 3, 4, 5). VSC production positively correlated with biomass, confirming the biological origin of VSCs (Fig. 1). Although no significant decline of l-methionine was observed (data not shown), the formation of VSCs remained stable after 48-h incubation, suggesting l-methionine was not rate-limiting. Our results are consistent with the findings of Etschmann et al. (2008) and Tan et al. (2012), who reported that no significant increase of methionol was observed in broth supplemented with l-methionine fermented by *S. cerevisiae* and *Williopsis* yeasts.

Aeration significantly affected the production of VSCs. The production of methionol was almost 12 times higher under static condition than with agitation (Fig. 3). This is expected as methionol formation is a reaction of reduction from methional catalyzed by alcohol dehydrogenase and is usually produced under oxygen-limited conditions (Tan et al. 2012). Our results differed from the finding of Hébert et al. (2011), where the *K. lactis* exponential growing cells did not produce any methionol under their condition; this could be due to the different cell states (non-growing cells of *K. lactis* used in this study). The decreased production of methionol under aeration could be related to repression of pyruvate decarboxylase and alcohol dehydrogenase activities by high oxygen levels (Landaud et al. 2008; Miyakoshi et al. 2016; Tan et al. 2012; Ugliano and Henschke 2009), since both enzymes are thought to be responsible for methionol formation through l-methionine catabolism (Miyakoshi et al. 2016; Tan et al. 2012). *K. lactis* is a respiro-fermentative yeast, it produces little or no alcohols under very high aeration, especially when sugar is limited (Becerra et al. 2004). In this study, however, no sugar was added and aerobic respiration was the key pathway of glucose (residual) conversion into pyruvate via glycolysis, and then to acetyl-CoA and CO_2_ via citric acid cycle (González-Siso et al. 2009).

In contrast, aeration significantly boosted MTA and DMDS formation initially, especially at 80 rpm, suggesting increased production of l-methionine-derived methanethiol (a precursor common to both MTA and DMDS). This could be due to the respiro-fermentative nature of *K. lactis* that promoted the catabolism of l-methionine by aeration. Methanethiol then reacted with acetyl-CoA to form MTA or was oxidized to produce DMDS. On the other hand, since DMDS is an oxidized product of methanethiol and a lack of oxygen under static condition could inhibit the oxidation reactions, resulting in minor DMDS formation (Bonnarme et al. 2004). Nonetheless, for all analyzed VSCs, vigorous agitation could promote the volatilization of VSCs and their losses.

The highest levels of methionol and MTA were produced at 30 °C (Fig. 4). However, the production of methionol was not significantly different among all treatments except 38 °C for MTA, which might indicate that temperature did not completely inhibit their formation, but partially inactivated the enzyme activity. The lowest level of MTA (known to be formed enzymatically, at least partially) was produced at 38 °C, which might be due to the cell death of *K. lactis* (Additional file 1: Table S2). Helinck et al. (2000) investigated the effect of temperature on the production of MTA in *G. candidum* with the addition of methanethiol and acetyl-CoA and found that more MTA formation at higher temperatures (45 and 56 °C). By comparison, in our study, the highest level of DMDS was produced at 38 °C, which was inversely correlated with the lowest formation MTA at the same temperature. This negative correlation could be attributed to the exacerbation of thermally induced disulfide bond formation via oxidation of methanethiol as a result of diminished MTA formation (i.e. reduction of methanethiol consumption) and the availability of methanethiol (the precursor to both DMDS and MTA).

The pH significantly affected the production of VSCs (Fig. 5). The cell counts at pH 4.0 declined sharply after incubation, then resulting in less production of VSCs. The highest production of VSCs was detected at pH 5.0, which is considered as the optimum pH for the growth of *K. lactis*. Similar results were reported by Liu and Crow (2010) and Seow et al. (2010).

### Effects of nutrient supplementation on kinetic changes of VSCs by non-growing cells of *K. lactis*

Nitrogen supplementation had a negative impact on the production of VSCs, as it reduced the formation of methionol, MTA and DMDS in comparison to non-supplemented samples (Fig. 6). (Hernández-Orte et al. 2005) investigated the addition of amino acids and ammonium salts to must for wine making and found that methionol produced from the ammonium addition was significantly reduced. Similar results were reported by Tan et al. (2012), who showed that a statistically significant decrease (*P *< 0.05) of VSC production with an increase of nitrogen supplementation (0.05, 0.10 and 0.20%). The inhibition of formation of methionol, MTA and DMDS by nitrogen supplementation may indicate a direct or indirect biological transformation of l-methionine to these VSCs, rather than pure chemical degradation (Tan et al. 2012). Ammonium is an easily assimilable nitrogen sources over other forms by yeasts (Clement et al. 2013; Tan et al. 2012). An excess of ammonium than cellular reserves will negate the need for the catabolism of l-methionine and therefore decreasing the production of VSCs (Bisson 1991; ter Schure et al. 2000; Tan et al. 2012).

A significantly positive correlation exists between yeast extract concentration and VSC including methional production (Fig. 7). Yeast extract is composed of readily assimilable nitrogen sources such as amino acids and the water-soluble vitamin B complex. The marked increase of methionol could be related to the vitamin B complex, which is considered to be an indispensable cofactor in many enzymatic reactions. The activity of aminotransferase (ATase) is PLP-dependent and PLP in turn arises from vitamin B_6_. The ARO8 gene coding the ATase involved in l-methionine degradation was identified in *K. Lactis* (Hébert et al. 2011). The crystal structure of *S. cerevisiae* Aro8 protein was determined and indicated the PLP cofactor binding (Bulfer et al. 2013). Therefore, due to the added amounts of vitamin B_6_, ATase activity increased and expedited l-methionine transamination and methionol production via the Ehrlich pathway. The boosted l-methionine catabolism and VSC production offset the nitrogen catabolite repression brought about by the amino acids from the yeast extract. The excretion of methional could be the result of accelerated rates of l-methionine transamination and α-keto-γ-methylthio-butyric acid (KMBA, which could be excreted from the cells) production (Hébert et al. 2011), such that the rate of KMBA decarboxylation to methional was greater than that of methional reduction to methionol. However, methional formation may also be partially ascribed to Strecker degradation of l-methionine, which could be accelerated by riboflavin (vitamin B_2_) present in the yeast extract.

Our results showed that Mn^2+^ could enhance the conversion of l-methionine into methionol. Mn^2+^ is a co-factor of aminotransferase and/or decarboxylase, or Mn^2+^ could regulate redox conditions. Given that no related studies were reported and this hypothesis needs further investigation. However, the present study showed that transition metals seemed to be toxic to yeast cells, causing the relatively sharp decline of yeast population in the presence of Mn^2+^ (Additional file 1: Table S2). Interestingly, Mn^2+^ decreased MTA formation but increased DMDS production. It is known that DMDS is formed from the oxidation of methanethiol, a reaction that is catalyzed by transition metals and thus, the higher amount of DMDS with the addition of Mn^2+^ could be the result of methanethiol oxidation accentuated by Mn^2+^ (Chin and Lindsay 1994). By the same token, this explains the lower production of MTA (i.e. due to methanethiol being diverted to DMDS). In addition, the production of DMDS is not obligatorily dependent on yeast viability but availability of methanethiol, oxygen and catalyst(s).

In conclusion, the present study investigated for the first time the VSCs producing feasibility in a model system consisting of non-growing cells of *K. lactis* and l-methionine. Several pathways are involved in VSC formation through whole-cell-based catabolism of l-methionine which can be manipulated by adjusting physicochemical parameters and nutrient supplementation. The whole-cell system has proven to be a useful catalytic system, enhancing the biosynthesis of some VSCs. This study showed the feasibility of exploiting metabolically active non-growing cells in non-growth media to produce a range of sulphur-bearing, natural flavors from only one precursor, l-methionine. These produced VSCs might be widely used in food and beverage industries due to their high potency in flavor contributions. The findings may facilitate development of whole cell-based systems to produce these VSCs on a larger scale.

## Additional file

**Additional file 1: Table S1.** Volatile sulphur compounds (Average FID peak area × 10^5^ ± SD) produced during 48 h incubation. **Table S2.** *K. lactis* cell count (CFU/mL × 10^7^) before (0 h) and after fermentation (48 h).

## Abbreviations

VSCs: volatile sulphur compounds
DMDS: dimethyl disulfide
DMTS: dimethyl trisulfide
MTA: *S*-methyl thioacetate
PDA: potato dextrose agar
MnCl_2_·4H_2_O: manganese (II) chloride tetrahydrate
NaH_2_PO_4_·2H_2_O: sodium phosphate monobasic dihydrate
Na_2_HPO_4_: sodium phosphate dibasic anhydrous
DAP: diammonium phosphate
OD: optical density
HS-SPME–GC–MS–FID: head space solid-phase micro extraction coupled with gas chromatography–mass spectrometry and flame ionization detection
ANOVA: one-way analysis of variance
KMBA: α-keto-γ-methylthio-butyric acid production
