# Supplementary material for: Effects of physicochemical parameters on volatile sulphur compound formation from l-methionine catabolism by non-growing cells of Kluyveromyces lactis
Source: AMB Express. 2018 Jul 3;8:109. doi: 10.1186/s13568-018-0639-7 (PMC6029988; doi:10.1186/s13568-018-0639-7)
Supplement: Supplementary file 1 — Additional file 1: Table S1. Volatile sulphur compounds (Average FID peak area × 105 ± SD) produced during 48 h incubation. Table S2. K. lactis cell count (CFU/mL × 107) before (0 h) and after fermentation (48 h). [file 13568_2018_639_MOESM1_ESM.docx]

**AMB Express**

**Additional file 1**

**Effects of physicochemical parameters on volatile sulphur compound formation from L-methionine catabolism by non-growing cells of *Kluyveromyces lactis***

Yuyun Lu^a^, Margarete Nawrath^a^, Jingcan Sun^a^, Shao-Quan Liu^a,b^*

^a^ Food Science and Technology Program, Department of Chemistry, National University of Singapore, Science Drive 3, Singapore 117543, Singapore

^b^ National University of Singapore (Suzhou) Research Institute, 377 Lin Quan Street, Suzhou Industrial Park, Jiangsu 215123, China

*Corresponding author. Postal address: Food Science and Technology Programme, Department of Chemistry, National University of Singapore, Science Drive 3, Singapore. Tel.: +65 6516 2687; fax: +65 6775 7895.

E-mail address: chmlsq@nus.edu.sg (Shao-Quan Liu)

**Table S1.** Volatile sulphur compounds (Average FID peak area × 10^5^ ± SD) produced during 48 h incubation

|  |  |  | **Methionol** | | **S-Methyl thioacetate** | | **DMDS** | | **Methional** | |
| --- | --- | --- | --- | --- | --- | --- | --- | --- | --- | --- |
| Runs | Parameters | Factor level | Peak Area (0 h) | Peak Area (48 h) | Peak Area (0 h) | Peak Area (48 h) | Peak Area (0 h) | Peak Area (48 h) | Peak Area (0 h) | Peak Area (48 h) |
| **1** | Biomass  (OD_600_) | 2 | 0.18±0.00^Aa^ | 4.52±0.07^Ba^ | 0.00±0.00^Aa^ | 1.91±0.37^Ba^ | 1.58±0.00^Aa^ | 7.61±0.66^Ba^ | 0.00±0.00^Aa^ | 0.00±0.00^Aa^ |
|  |  | 4 | 0.18±0.00^Aa^ | 5.01±0.64^Ba^ | 0.00±0.00^Aa^ | 9.23±1.10^Bb^ | 1.39±0.00^Aa^ | 13.44±1.76^Bb^ | 0.00±0.00^Aa^ | 0.00±0.00^Aa^ |
|  |  | 6 | 0.16±0.00^Aa^ | 10.22±2.34^Bb^ | 0.00±0.00^Aa^ | 21.23±5.79^Bb^ | 0.64±0.00^Aa^ | 21.02±1.64^Bc^ | 0.00±0.00^Aa^ | 0.00±0.00^Aa^ |
| **2** | L-methionine (w/v) | 0.1% | 0.00±0.00^Aa^ | 3.43±0.23^Ba^ | 0.00±0.00^Aa^ | 16.17±3.60^Ba^ | 0.90±0.10^Aa^ | 11.20±0.10^Ba^ | 0.00±0.00^Aa^ | 0.00±0.00^Aa^ |
|  |  | 0.5% | 0.00±0.00^Aa^ | 4.10±0.61^Ba^ | 0.00±0.00^Aa^ | 17.20±2.83^Ba^ | 1.28±0.07^Ab^ | 11.17±1.78^Ba,b^ | 0.00±0.00^Aa^ | 0.00±0.00^Aa^ |
|  |  | 1.0% | 0.00±0.00^Aa^ | 4.02±0.13^Ba^ | 0.00±0.00^Aa^ | 17.02±3.84^Ba^ | 1.22±0.15^Aa,b^ | 8.38±0.27^Bb^ | 0.00±0.00^Aa^ | 0.00±0.00^Aa^ |
| **3** | Agitation  (rpm) | 0 | 0.00±0.00^Aa^ | 11.26±0.80^Ba^ | 0.00±0.00^Aa^ | 3.75±0.27^Ba^ | 0.79±0.07^Aa^ | 6.16±0.44^Ba^ | 0.00±0.00^Aa^ | 0.00±0.00^Aa^ |
|  |  | 80 | 0.00±0.00^Aa^ | 2.83±0.16^Bb^ | 0.00±0.00^Aa^ | 6.24±1.41^Ba^ | 0.79±0.07^Aa^ | 9.45±0.39^Bb^ | 0.00±0.00^Aa^ | 0.00±0.00^Aa^ |
|  |  | 120 | 0.00±0.00^Aa^ | 0.94±0.13^Bc^ | 0.00±0.00^Aa^ | 3.91±0.31^Ba^ | 0.79±0.07^Aa^ | 4.63±0.35^Bc^ | 0.00±0.00^Aa^ | 0.00±0.00^Aa^ |
| **4** | Temperature  (°C) | 25 | 0.23±0.00^Aa^ | 5.54±0.44^Ba^ | 0.24±0.03^Aa^ | 3.33±0.56^Ba^ | 0.23±0.05^Aa^ | 2.24±0.08^Ba^ | 0.00±0.00^Aa^ | 0.00±0.00^Aa^ |
|  |  | 30 | 0.23±0.00^Aa^ | 7.51±1.58^Ba^ | 0.24±0.03^Aa^ | 5.03±0.26^Ba^ | 0.23±0.05^Aa^ | 3.27±0.43^Ba^ | 0.00±0.00^Aa^ | 0.00±0.00^Aa^ |
|  |  | 38 | 0.23±0.00^Aa^ | 5.97±0.27^Ba^ | 0.24±0.03^Aa^ | 0.26±0.00^Ab^ | 0.23±0.05^Aa^ | 9.74±0.85^Bb^ | 0.00±0.00^Aa^ | 0.00±0.00^Aa^ |
| **5** | pH | 4 | 0.00±0.00^Aa^ | 3.84±1.12^Ba^ | 0.00±0.00^Aa^ | 1.33±0.10^Ba^ | 0.00±0.00^Aa^ | 2.98±0.87^Ba^ | 0.00±0.00^Aa^ | 0.00±0.00^Aa^ |
|  |  | 5 | 0.00±0.00^Aa^ | 8.15±0.79^Bb^ | 0.00±0.00^Aa^ | 5.26±0.76^Bb^ | 0.00±0.00^Aa^ | 5.55±0.69^Bb^ | 0.00±0.00^Aa^ | 0.00±0.00^Aa^ |
|  |  | 6 | 0.00±0.00^Aa^ | 6.41±0.10^Ba,b^ | 0.00±0.00^Aa^ | 3.65±0.78^Ba,b^ | 0.00±0.00^Aa^ | 4.73±0.67^Ba,b^ | 0.00±0.00^Aa^ | 0.00±0.00^Aa^ |
| **6** | Nitrogen supplement  (w/v) | 0% | 0.00±0.00^Aa^ | 8.05±0.52^Ba^ | 0.00±0.00^Aa^ | 3.83±0.31^Ba^ | 0.00±0.00^Aa^ | 2.71±0.66^Ba^ | 0.00±0.00^Aa^ | 0.00±0.00^Aa^ |
|  |  | 0.1% | 0.00±0.00^Aa^ | 5.22±0.91^Ba,b^ | 0.00±0.00^Aa^ | 2.73±0.20^Bb^ | 0.00±0.00^Aa^ | 2.21±0.36^Ba^ | 0.00±0.00^Aa^ | 0.13±0.05^Bb^ |
|  |  | 0.3% | 0.00±0.00^Aa^ | 2.72±0.22^Bb^ | 0.00±0.00^Aa^ | 1.81±0.36^Bb^ | 0.00±0.00^Aa^ | 2.45±0.14^Ba^ | 0.00±0.00^Aa^ | 0.20±0.02^Bc^ |
| **7** | Yeast extract supplement  (w/v) | 0% | 0.30±0.02^Aa^ | 6.61±0.15^Ba^ | 0.00±0.00^Aa^ | 3.83±0.72^Ba^ | 0.00±0.00^Aa^ | 2.80±0.07^Ba^ | 0.00±0.00^Aa^ | 0.00±0.00^Aa^ |
|  |  | 0.1% | 0.79±0.14^Ab^ | 25.32±0.60^Bb^ | 0.21±0.02^Aa^ | 5.18±0.20^Ba^ | 0.00±0.00^Aa^ | 2.99±0.08^Ba^ | 0.00±0.00^Aa^ | 0.31±0.05^Bb^ |
|  |  | 0.3% | 1.54±0.14^Ac^ | 40.59±2.37^Bc^ | 0.34±0.03^Aa^ | 4.70±0.58^Ba^ | 0.00±0.00^Aa^ | 2.93±0.24^Ba^ | 0.26±0.09^Aa^ | 0.75±0.10^Bc^ |
| **8** | Mn^2+^ supplement  (mM) | 0 | 0.14±0.10^Aa^ | 7.59±0.34^Ba^ | 0.00±0.00^Aa^ | 4.42±0.79^Ba^ | 0.00±0.00^Aa^ | 3.18±0.20^Ba^ | 0.00±0.00^Aa^ | 0.00±0.00^Aa^ |
|  |  | 1 | 0.32±0.02^Aa^ | 9.23±0.43^Ba^ | 0.00±0.00^Aa^ | 3.24±0.14^Bb^ | 0.74±0.25^Aa^ | 6.18±0.24^Bb^ | 0.00±0.00^Aa^ | 0.00±0.00^Aa^ |
|  |  | 10 | 0.65±0.15^Aa^ | 9.54±0.61^Ba^ | 0.00±0.00^Aa^ | 2.83±0.32^Bb^ | 0.83±0.19^Aa^ | 6.29±0.34^Bb^ | 0.00±0.00^Aa^ | 0.00±0.00^Aa^ |

^A,B^ Statistical analysis at 95% confidence level with same letters within same row of the same compounds indicating no significant difference. #6, nitrogen = diammonium phosphate.

^a,b,c^ Statistical analysis at 95% confidence level with same letters within column of the same compounds in the respective parameter indicating no significant difference.

**Table S2.** *K. lactis* cell count (CFU/mL × 10^7^) before (0 h) and after fermentation (48 h).

|  |  |  | **Yeast cell count (0 h)** | **Yeast cell counts (48 h)** | | |
| --- | --- | --- | --- | --- | --- | --- |
| Runs | Parameters | Factor level |  | **2** | **4** | **6** |
| **1** | Biomass  (OD_600_) | 2 | 3.63±1.01^a^ | 2.35±0.05^b^ |  |  |
|  |  | 4 | 7.30±0.86^a^ |  | 3.90±1.02^b^ |  |
|  |  | 6 | 11.30±1.56^a^ |  |  | 5.73±1.47^b^ |
| **2** | L-methionine(w/v) |  |  | **0.1%** | **0.5%** | **1.0%** |
|  |  |  | 13.80 ± 1.74^a^ | 11.00±0.29^b^ | 10.40±0.29^b^ | 10.77±0.45^b^ |
| **3** | Agitation (rpm) |  |  | **0** | **80** | **120** |
|  |  |  | 17.37 ± 0.67^a^ | 12.50±1.10^b^ | 5.07±0.90^c^ | 12.63±1.11^b^ |
| **4** | Temperature (°C) |  |  | **25** | **30** | **38** |
|  |  |  | 15.77 ± 0.75^ab^ | 19.57±2.65^b^ | 13.10±2.67^b^ | 0.00±0.00^c^ |
| **5** | pH |  |  | **4** | **5** | **6** |
|  |  |  | 14.14 ± 0.54^a^ | 4.97±0.29^b^ | 8.60±1.79^c^ | 8.83±0.54^c^ |
| **6** | Nitrogen supplement (w/v) |  |  | **0%** | **0.1%** | **0.3%** |
|  |  |  | 10.63 ± 2.20^a^ | 8.53±1.18^a^ | 10.45±0.35^a^ | 7.93±3.11^a^ |
| **7** | Yeast extract  supplement (w/v) |  |  | **0%** | **0.1%** | **0.3%** |
|  |  |  | 9.64 ± 1.41^a^ | 9.10±3.72^a^ | 9.87±1.07^a^ | 11.37±1.62^a^ |
| **8** | Mn^2+^ supplement (mM) |  |  | **0** | **1** | **10** |
|  |  |  | 15.82 ± 1.48^a^ | 9.90±1.79^b^ | 7.40±1.34^bc^ | 4.33±0.60^c^ |

^a, b, c^ Statistical analysis at 95% confidence level with same letters within line indicating no significant difference. #6, nitrogen = diammonium phosphate.
